# Genetic–geographic correlation revealed across a broad European ecotypic sample of perennial ryegrass (*Lolium perenne*) using array-based SNP genotyping

**DOI:** 10.1007/s00122-015-2556-3

**Published:** 2015-06-21

**Authors:** T. Blackmore, I. Thomas, R. McMahon, W. Powell, M Hegarty

**Affiliations:** Institute of Biological, Environmental and Rural Sciences, Aberystwyth University, Gogerddan, Aberystwyth, Ceredigion SY23 3EE Wales, UK

## Abstract

****Key message**:**

**Publically available SNP array increases the marker density for genotyping of forage crop,*Lolium perenne*. Applied to 90 European ecotypes composed of 716 individuals identifies a significant genetic–geographic correlation.**

**Abstract:**

Grassland ecosystems are ubiquitous across temperate and tropical regions, totalling 37 % of the terrestrial land cover of the planet, and thus represent a global resource for understanding local adaptations to environment. However, genomic resources for grass species (outside cereals) are relatively poor. The advent of next-generation DNA sequencing and high-density SNP genotyping platforms enables the development of dense marker assays for population genetics analyses and genome-wide association studies. A high-density SNP marker resource (Illumina Infinium assay) for perennial ryegrass (*Lolium perenne*) was created and validated in a broad ecotype collection of 716 individuals sampled from 90 sites across Europe. Genetic diversity within and between populations was assessed. A strong correlation of geographic origin to genetic structure was found using principal component analysis, with significant correlation to longitude and latitude (*P* < 0.001). The potential of this array as a resource for studies of germplasm diversity and identifying traits underpinning adaptive variation is highlighted.

**Electronic supplementary material:**

The online version of this article (doi:10.1007/s00122-015-2556-3) contains supplementary material, which is available to authorized users.

## Introduction

Grassland ecosystems account for approximately 40 % of the terrestrial land mass of our planet and are of critical importance to carbon sequestration, the bio-geochemistry of soils and the maintenance of biodiversity (Tilman et al. 1996; Jones and Donnelly 2004). Perennial ryegrass (*Lolium perenne* L.) is a dominant species of temperate grassland ecosystems, covering a broad range of environmental conditions (day length, moisture, altitude, soil type and chemistry, etc.). Understanding the patterns and magnitude of genetic diversity in the allogamous forage grass species *L. perenne* is thus a useful first step towards identifying loci under selection for multiple ecological traits, and also serves as a gateway for gene discovery in other grasses, with which it shares considerable synteny (Jones et al. 2002). To date, genomic resources in *Lolium* have been relatively poor, but NGS is rapidly facilitating the development of high-density marker assays, such as the Illumina GoldenGate assay developed by Studer et al. (2012).

The genetic diversity in wild populations (ecotypes) has previously been studied in *L. perenne* (Balfourier et al. 1998, 2000; Bolaric et al. 2005a, b; Cresswell et al. 2001; McGrath et al. 2007; Skot et al. 2005; Yu et al. 2011). These have all used techniques, such as AFLP, RFLP and RAPD, whereby only a low marker density was assayed and/or a limited number of populations surveyed. QTLs have been discovered in ecotypic populations for commercially important traits, such as heading date (with its association to digestibility) and submergence resistance (Skot et al. 2005; Yu et al. 2011), demonstrating that these natural populations offer opportunities to discover new marker/trait associations.

Studies of natural populations are increasingly turning towards high-density, genome-wide approaches to understanding genetic diversity (Brumfield et al. 2003; Garvin et al. 2010). The reasons for this are threefold: firstly, because such approaches provide extra resolution over older marker technologies—enabling fine-scale changes in population structure and/or history to be uncovered (Luikart et al. 2003; Morin et al. 2009). Secondly, these technologies lend themselves readily to association genetics studies of complex adaptive traits (Syvänen 2001) and, finally, due to the relative ease with which these assays can be established (Vignal et al. 2002). The advent of next-generation DNA sequencing (NGS) has enabled researchers to rapidly access genome-wide information for their study organism, regardless of whether a full genome sequence exists (Kircher and Kelso 2010; Morozova and Marra 2008). This provides a rich resource which can be mined for genetic markers—thousands to millions of single nucleotide polymorphisms (SNPs) can be putatively identified in silico for a modest outlay in NGS coverage. With access to high-density SNP genotyping technologies, these markers can be used to screen large populations at a genome-wide level in timeframes which would be impossible with other markers such as SSRs or AFLPs (Brumfield et al. 2003; Willing et al. 2010). The genomic abundance and amenability to cost-effective high-throughput genotyping have meant that SNPs are developing into the most widely used class of genetic marker in the analysis and dissection of inherited complex traits, particularly those that contribute to adaptive, ecological variation (Bergelson and Roux 2010).

SNPs can be utilised using different methods: direct sample sequencing with techniques such as restriction site associated DNA sequencing (RAD; Baird et al. 2008) or genotyping by sequencing (GBS; Elshire et al. 2011) or by SNP array platforms. Each technique has its advantages which are applicable depending on the experimental design and overall aim (Thomson 2014). With the falling costs of sequencing, barcoding samples for NGS sequencing allows an accessible method of SNP genotyping with no prior sequence knowledge or reference genome. However, the bioinformatic analysis has greater demands in terms of pipeline integration and in computing power and storage capacity for the generated data. Furthermore, the reduced representational libraries in the form of RAD tags and GBS are heavily dependent on imputation to fill missing data (Huang et al. 2009). In contrast, once the initial sequencing, probe selection and marker validation has resulted in the creation of an SNP array, array-based genotyping provides a reproducible technique across users and laboratories. Sequencing-based methods are also often prone to loss of shared loci across experiments, whilst array-based markers perform relatively consistently (though individual markers may be monomorphic or null in given populations). The resulting genotypes are thus easy to compare to previous data and experiments due to the same SNPs being typed. Unlike NGS techniques, the analysis of array platform data is possible with a desktop computer with minimal memory/storage requirements.

We report here on the creation and validation of a publically available custom Illumina Infinium SNP genotyping microarray for *L. perenne* represented by 2185 validated SNP markers and its application to screening a large European ecotype population of over 700 individuals. We assess the population structure of this collection and note the strong correlation of genotype to geographic origin, which suggests the value of this array for studies of population genetics and adaptive trait variation in ryegrass.

## Materials and methods

### Next-generation sequencing

To identify putative SNP loci which could be used to construct an Infinium assay, we conducted Illumina RNAseq of five diverse genotypes of *L. perenne* which were contributed as clonal replicates (tillers) by the researchers referenced below. The five genotypes selected were: AberMagic (an IBERS synthetic forage variety, R. Hayes, pers. comm.); a Chromosome 3 substitution line with *Festuca pratensis* (King et al. 2002); a mother plant from the IBERS late heading recurrent breeding population (R. Hayes, pers. comm.); a “stay-green” amenity variety (Thorogood et al. 1993) and an early flowering ecotypic sample from France previously described in Skøt et al. (2007). These genotypes thus represent a selection of *L. perenne* from wild to highly selected “domesticated” lines. As we were not concerned with gene expression (only SNP detection), a single individual was grown for each genotype. Each individual was harvested at the young (3–4 weeks post-germination) stage and total RNA isolated from both total above and below ground biomass using Trizol extraction (Sigma Aldrich). The above/below ground extracts were pooled for each individual genotype at equimolar concentrations prior to Illumina RNAseq library construction, to provide as much coverage of the transcriptome at equivalent life history stages (flowering tissue was ignored as the genotypes used display significant variation). Aliquots of 2 µg of total RNA per genotype were used to prepare libraries as per the Illumina mRNA-seq protocol (mRNA-Seq 8-sample Prep Kit (RS-100-0801). Each library was sequenced in a single lane of an Illumina GA-IIx platform at GenePool (University of Edinburgh) using paired-end 2 × 56 bp sequencing. Read count averaged 41 million reads per genotype (20.5 million pairs), with the lowest output being the amenity genotype with 13 million pairs and the highest AberMagic (50.5 million pairs). Raw FASTQ data for these libraries are available through the NCBI short read archive (http://www.ncbi.nlm.nih.gov/sra), accessions SRR2034619–SRR2034623.

### Sequence assembly and SNP detection

Reads were imported into the Genomics Workbench version 4.5.1 package (CLC Bio Ltd.) and a reference transcriptome was assembled de novo using the reads from AberMagic, since it generated the highest read coverage. De novo assembly in Genomics Workbench uses the de Bruijn graph method with a k-mer value assigned based on the scale of data input (for 2.75 Gbp as here, a k-mer of 23 is assigned). The maximum bubble size for conflict resolution within the graph was set at 50. Repeat regions within the graph were resolved using scaffolding based on paired-end sequences. Following initial contig assembly, reads were mapped back to contigs, requiring 50 % match at 80 % similarity across the read. Ambiguous read mappings (reads mapping to more than one contig) were discarded from the mapping. Insertion and deletion penalties were set at 3 and mismatch penalty at 2. Contigs from the initial assembly were removed if no reads mapped. This step was included to resolve conflicts by generating a consensus based on the most common base for each position.

This assembly produced a total of 55,181 contigs which were used as the reference for read mapping of the five genotypes. This Transcriptome Shotgun Assembly project has been deposited at DDBJ/EMBL/GenBank under the accession GDAT00000000. The version described in this paper is the first version, GDAT01000000. BLASTx annotation of contigs (Altschul et al. 1990) was performed within the Genomics Workbench package using a local copy of the non-redundant (nr) protein database (downloaded circa August 2011). Individual mappings were produced for each genotype (as above, but employing 50 % match at 95 % identity across each read), which were then mined for the presence of SNPs. Non-specific read mappings (reads mapping to >1 contig) were ignored (to avoid identification of SNPs within multigene families), and a minimum quality score of 20 was requested surrounding the putative SNP (quality score for the SNP itself was requested as 30 or higher). To further increase stringency and avoid issues with sequence error, a minimum read coverage of 50 was requested for each SNP. Minor allele variant detection threshold was set at 25 % for similar reasons. Despite the stringency of these criteria, a total of 53,149 putative SNPs (within 11,892 unique contigs) were identified across the five genotypes.

### Infinium assay design

Despite the high number of putative SNPs identified, not all the putative SNPs identified were suitable for construction of Infinium probes: firstly, we needed to maximise the likelihood that markers would be informative across a broad range of material. Secondly, we needed to account for the possibility of misassembly during the de novo contig construction and remove sequences which might be present in high copy number. To address the first issue, we subselected markers which showed evidence of polymorphism in two or more of the accessions, reducing the possibility that a particular marker might not show polymorphism in wider *L. perenne* collections. For example, whilst we included the chromosome substitution line of King et al. (2002) because of its relevance to IBERS breeding programmes, the *Festuca* material might otherwise contribute a significantly higher number of polymorphisms (though in the event, this material showed a similar number of variants to AberMagic itself, with the natural *L. perenne* ecotype displaying the most polymorphism).

With regard to the possibility of misassembly in the contig data, we, therefore, excluded any contigs displaying evidence of frameshift (multiple hits to the same match) within their BLASTx result. Further filtering on BLAST identifier was then applied to remove likely organellar or retroelement sequences (as suggested by Illumina), which are likely to be present in high copy number or overrepresented in DNA extracts used for genotyping.

Finally, a minimum flanking sequence of 50 bp is required around the SNP for Infinium probe design (60 bp preferred), which would exclude some SNPs positioned close to the ends of contigs. Although Infinium technology is more tolerant of the presence of other SNPs within the probe sequence, we decided to err on the side of caution and also eliminate any SNPs within 50 bp of each other. Custom PERL scripts were designed to mine the contig FASTA file based on the SNP report tables produced by Genomics Workbench and isolate SNPs with sufficient flanking sequence which were >50 bp away from any other SNP. These filters reduced the number of possible SNPs to 4513 (spread across 2943 contigs). A custom PERL script was then employed to extract the flanking 50–60 bp around each marker and annotate the SNP itself with the format [allele1/allele2]. This provisional SNP probe set was uploaded to the Illumina Assay Design Tool (ADT) and the SNPs assessed for probe designability. SNPs with designability scores of 0.6 or higher were selected for inclusion in the final array design, producing an initial assay of 3775 putative SNPs in total. Subsequent validation steps (described below) reduced the final marker set to 2185 SNPs.

### Plant material

A bi-parental mapping population (Hegarty et al. 2013), consisting of 193 progeny and two parents (AberMagic × Aurora), was selected to use as a basis of marker validation via allele heritability. In addition, six progeny and two parental replicates were included to assess genotyping error rate.

The ecotype collection used for array validation was formed from *L. perenne* seed collected at various sites across Europe (Table 1) and subsequently germinated. Accessions were selected from an existing seedbank kept at IBERS, Aberystwyth, in order to represent a range of geographical locations (latitude, longitude and altitudes) as well as environments and land management conditions. Plants from each accession were allowed to polycross to bulk seed for each location. Plants and seed were maintained at IBERS, Aberystwyth University. Leaf tissue was harvested from individual *Lolium* plants and DNA was extracted using QIAGEN 96 plant tissue extraction kit. A total of 716 individual *L. perenne* ecotypes from a range of locations and environments across Europe were used, with 8 individuals within each of 89 accessions and four individuals from one accession.

**Table 1:** Geographic location of sample site for each accession

ID	Accession	Country	Longitude (°)	Latitude (°)	Altitude (MASL)
AT1	Ba10985	Austria	14.07	48.28	310
BG1	Ba12019	Bulgaria	24.78	42.85	525
BG2	Ba12020	Bulgaria	24.78	42.85	600
BG3	Ba12028	Bulgaria	26.18	42.90	490
BG4	Ba12039	Bulgaria	23.35	42.62	760
BG5	Ba12049	Bulgaria	22.48	42.22	1060
CH1	Ba10282	Switzerland	7.68	47.37	1120
CH2	Ba10284	Switzerland	8.85	47.44	720
CH3	Ba10286	Switzerland	8.93	47.28	1200
CH4	Ba10288	Switzerland	7.77	46.40	1840
CH5	Ba9101	Switzerland	7.38	46.18	2030
CH6	Ba9105	Switzerland	8.85	47.43	600
CZ1	Ba11862	Czech_Republic	17.85	49.45	380
CZ2	Ba11865	Czech_Republic	18.10	49.48	500
CZ3	Ba11869	Czech_Republic	17.98	49.67	240
CZ4	Ba11878	Czech_Republic	18.03	49.47	280
Eng1	Ba10015	England	−1.26	51.75	57
Eng2	Ba10292	England	0.76	50.96	0
Eng3	Ba11141	England	−0.14	52.84	0
Eng4	Ba11143	England	−0.67	52.77	120
Eng5	Ba13209	England	−2.87	51.02	30
Eng6	Ba13228	England	−2.33	54.77	550
Eng7	Ba13240	England	−2.82	51.30	230
Eng8	Ba13241	England	−2.77	51.28	100
Eng9	Ba9960	England	−2.81	51.23	0
ES1	Ba13697	Spain	−0.18	42.66	1734
ES2	Ba13698	Spain	−0.17	42.62	1245
ES3	Ba13705	Spain	−0.30	42.72	1092
ES4	Ba13706	Spain	−0.42	42.80	1760
ES5	Ba13724	Spain	−0.61	42.33	1075
ES6	Ba13735	Spain	−0.02	42.57	1299
ES7	Ba13740	Spain	−0.53	42.68	982
ES8	Ba13858	Spain	−5.85	43.20	857
ES9	Ba13859	Spain	−5.91	43.17	1535
ES10	Ba13860	Spain	−5.90	43.17	1373
ES11	Ba13867	Spain	−5.89	43.16	895
ES12	Ba13874	Spain	−7.01	43.35	877
ES13	Ba13876	Spain	−6.22	42.58	1194
ES14	Ba13877	Spain	−7.00	42.73	1229
ES15	Ba13882	Spain	−6.17	42.85	1253
ES16	Ba13884	Spain	−6.40	42.97	1379
ES17	Ba13885	Spain	−5.87	43.38	374
ES18	Ba13892	Spain	−5.61	43.18	851
FR1	Ba9109	France	6.13	48.30	287
GR1	Ba11900	Greece	20.78	39.55	NA
HU1	Ba11311	Hungary	20.58	46.85	NA
IE1	Ba10127	Ireland	−8.75	53.29	100
IE2	Ba10148	Ireland	−8.29	51.79	50
IE3	Ba10153	Ireland	−9.68	51.41	2
IE4	Ba10162	Ireland	−9.44	51.68	NA
IE5	Ba10170	Ireland	−9.50	52.06	20
IE6	Ba10178	Ireland	−8.34	54.68	40
IT1	Ba13445	Italy	12.55	46.32	800
IT2	Ba13448	Italy	13.50	45.85	100
IT3	Ba13457	Italy	12.92	46.08	250
IT4	Ba13458	Italy	12.80	45.83	100
IT5	Ba13463	Italy	13.15	45.75	1
IT6	Ba13470	Italy	11.77	45.55	100
IT7	Ba8590	Italy	7.58	45.02	270
IT8	Ba8596	Italy	7.47	44.33	700
IT9	Ba8617	Italy	10.29	46.49	1846
IT10	Ba8622	Italy	7.04	45.14	300
IT11	Ba11902	Italy_Sardegna	9.37	40.22	1000
NL1	Ba9246	Netherlands	7.03	53.12	0
NO1	Ba10103	Norway	5.67	58.72	50
NO2	Ba10111	Norway	5.33	59.92	10
NO3	Ba10113	Norway	6.63	61.18	75
PL1	Ba11427	Poland	20.95	51.68	100
PL2	Ba11429	Poland	20.65	50.85	300
PL3	Ba11431	Poland	20.67	50.87	270
PL4	Ba11449	Poland	20.57	49.42	700
PL5	Ba11453	Poland	20.30	49.40	500
PT1	Ba13099	Portugal	−6.82	41.88	841
PT2	Ba13101	Portugal	−6.98	41.80	444
PT3	Ba13104	Portugal	−7.78	41.82	1133
PT4	Ba13132	Portugal	−9.15	39.37	28
RO1	Ba9971	Romania	26.33	47.45	350
RO2	Ba9984	Romania	21.82	46.98	100
RO3	Ba9990	Romania	25.80	45.85	600
Sct1	Ba14025	Scotland	−7.52	57.60	15
Sct2	Ba14026	Scotland	−8.56	57.81	10
Sct3	Ba14053	Scotland	−6.03	57.22	5
SK1	Ba11887	Slovakia	19.42	48.82	650
TR1	Ba9123	Turkey	42.04	41.12	1210
TR2	Ba9151	Turkey	30.39	40.78	110
Wal1	Ba10951	Wales	−3.63	52.61	180
Wal2	Ba12142	Wales	−4.68	52.12	40
Wal3	Ba14027	Wales	−4.05	52.51	0
Wal4	Ba9791	Wales	−4.08	52.42	100
Wal5	Ba9799	Wales	−3.78	51.90	100

### Genotyping and assay validation

Genotyping was performed as per the manufacturer’s guidelines using the Illumina Infinium iSelect custom assay (Illumina, San Diego, CA, USA). There was a 91 % assay conversion rate resulting in 3425 putative SNPS on the final array (2334 in unique contigs). The *L. perenne* ecotype population of 716 individual plants (in addition to five randomly selected replicates) was genotyped using the custom Infinium assay and the data used to produce a cluster file for allele calling. Clustering was initially performed using automated cluster assignment within Illumina’s Genome Studio software. However, comparison of the clustering of the SNPs was inconsistent and manual reassignment of cluster position was required. This was independent of the original GenTrain score and, therefore, we were unable to manually reassign the cluster position to only SNPs with a GenTrain score below a certain threshold. Therefore, although laborious, all SNPs were visually inspected by one person for their original automated AA/AB/BB cluster positions and manually reassigned where appropriate. This also included the exclusion of SNPs with poor performance in this genetically diverse population. Markers were excluded where the average intensity (*R* mean) for each cluster was below 0.2 or cluster separation was less than 0.3. Markers were reviewed where cluster separation ranged between 0.3 and 0.45 (guidance from clustering algorithm metrics from Illumina). Markers were also excluded where there were missing data for more than 10 % of 716 samples, leaving 2501 markers at this stage. The wide range of genotypes used at this stage will maximise the general utility of the selected probes for further studies, because any interference due to genetic polymorphism resulting from genetic distance between the sequenced plants and the tested individuals will lead to exclusion from the array at this stage.

Although the original 5 individuals that were sequenced for the identification of SNPs were genotyped on the array, the comparison of the RNA sequence data to the genomic DNA SNP array proved difficult due to lack of information on allelic expression bias. Therefore, to further validate the markers, the cluster positions of the 2501 markers on the ecotype samples were exported and applied to the biparental mapping population that had also been genotyped on the array. This enabled use of heritability of alleles within a segregating population to be employed as confirmation of marker behaviour. Of the 2501 markers, 43 markers had more than four parent–parent–child heritability errors and were subsequently excluded, leaving a total of 2458 markers for further analysis on the ecotype population.

Thus, following reassignment of cluster positions or exclusion of markers using all 716 samples 2458 loci were exported. 239 had a minor allele frequency less than 5 % and were, therefore, excluded. Markers were also tested for observed heterozygosity (Ho) excess using GenePop (Raymond and Rousset 1995) in each of the 90 accessions. 34 markers with a probability less than 0.5 for Ho excess were also excluded to minimise genotyping errors. Following these exclusion parameters, a final validated set of 2185 SNP markers (spanning 1606 unique contigs) was available and used to assess the genetic diversity in the ecotype panel. Marker details have been uploaded to dbSNP (http://www.ncbi.nlm.nih.gov/SNP/) under accessions ss1751856902–ss1751859086 and are due for public release in Autumn 2015. Probe details are thus also provided in Supplementary Data Table 2. Marker names follow the convention “ContigX_Y” where X is the contig number as in the NCBI Transcriptome Shotgun Assembly (accession GDAT00000000) and Y is the base position of the SNP within that contig. Users may freely employ these probes in their own assays; alternatively IBERS offer access to the existing array as a genotyping service (contact corresponding author).

### Genetic diversity analysis

Data exported from Genome Studio (Illumina) were converted to allele specific presence. For the A alleles for each SNP, AA individuals were coded as 1, AB as 0.5 and BB and missing data were 0, and vice versa for the B alleles. Missing data were, therefore, coded as 0 for both the A and B alleles and were, therefore, not imputed. Allele frequencies for each marker within accessions were calculated by summing the values for the genotypes (as described above) for each individual within an accession for each SNP and divided by the number of individuals in the accession. Principal component analysis (PCA) was performed on these relative allele frequencies using R (version 2.15.3). Markers contributing the most to PC1 and PC2 were identified via the absolute loading of each marker to the respective PC. For each PC, the BLASTx annotation for the top 50 markers was investigated (Tables 3, 4).

Diversity measures were calculated within each of the accessions using GenAlEx (Peakall and Smouse 2006). Distribution of variation between geographic regions (as observed and defined following PCA and Supplementary Fig. 1) between accessions and within accessions was calculated using AMOVA within GenAlEx. This was reported as percentage of variation and measures of Phi_PT_. Phi_PT_ is used for codominant data as it suppresses intra-individual variation (Teixeira et al. 2014). AMOVA between neighbouring regions was also performed, treating each region as a single population (1 df) to compare to a previous study speculating at the divergence pattern and migration of *L. perenne* across Europe (McGrath et al. 2007).

Population structure was inferred using an unbiased Bayesian approach Markov chain Monte Carlo (MCMC) clustering of samples via STRUCTURE v2.3.4 (Pritchard et al. 2000). The data were assessed for prior values of *K* ranging from 1 to 10 with burnin and MCMC iterations settings at 25,000 and 25,000, respectively. For each value of *K*, 3 replications were performed. STRUCTURE Harvester v.0.6.93 was then used to identify the optimal value of *K* (using Δ*K* value; second-order rate of change in log probability between successive values of *K*) (Earl and VonHoldt 2012) with CLUMPP used to generate a consensus between runs (Supplementary Fig. 2). Probability of individual membership to group 1 was used to correlate with longitude of sample site origin.

## Results

### Performance of the array

The *Lolium* Infinium beadchip assayed 2185 markers with call rates that exceeded 99 % in 86 out of 90 ecotype accessions. The remaining four accessions had average call rates ranging from 97.8 to 98.7 %. As these call rates were consistent between individuals in the accession and across sample replicates, these data were included. Reproducibility of sample replicates was extremely high, with accuracy greater than 99.9 %.

### Genetic diversity

The Infinium platform was used to quantify the diversity present in 90 geographically referenced ecotype accessions, represented by 716 individual genotypes, spanning 21 countries and across a range of geographical conditions in Europe (Table 1). As seed for each sample site was germinated and polycrossed within accession at Aberystwyth seed bank, the individuals from each accession would be expected to display greater observed heterozygosity than expected under normal population genetics assumptions due to the self-incompatibility complex in *L. perenne*. The allele frequencies across individuals in an accession (population), however, are representative of those in the sampled location. Allele frequency was, therefore, used to represent the sample locations across Europe in analyses.

**Fig. 1:**
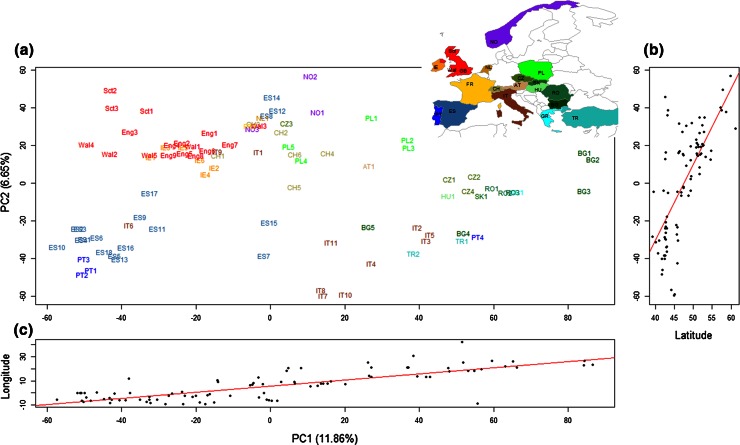
Principal component analysis of the allele frequencies of 90 ecotype accessions (with eight individuals) from 20 countries based on PC1 and PC2. **a** PC1 v PC2; *small coloured labels* represent each accession. *Inset map* provides key to labels. *AT* Austria, *BG* Bulgaria, *CH* Switzerland, *CZ* Czech Republic, *ES* Spain, *FR* France, *Eng* England, *GR* Greece, *HU* Hungary, *IE* Ireland, *IT* Italy, *NL* Netherlands, *NO* Norway, *PL* Poland, *PT* Portugal, *RO* Romania, *Sct* Scotland, *SK* Slovakia, *TR* Turkey, *Wal* Wales. **b** Correlation plot of PC2 with latitude of original seed sample site. Correlation coefficient, *R* ^2^ = 0.712, *P* < 0.0001. **c** Correlation plot of PC1 with longitude of original seed sample site. Correlation coefficient, *R* ^2^ = 0.798, *P* < 0.0001

The distribution of the genetic variation was also considered and partitioned based on the outcome of the PCA and geographic–genetic correlations (see below; Fig. 1a; Supplementary Fig. 1). The variation was compared between four regions, between accessions within region and between individuals within accessions using Phi_PT_ (analogous to *F*_ST_). The regions were defined by groupings observed in the PCA plot (Supplementary Fig. 1). Whilst some genetic variation was partitioned between regions, the greatest diversity (68 %) was attributed to between individuals within an accession (Table 2). Phi_PT_ showed greater variation between populations within regions (Phi_PR_), compared to between regions (Phi_RT_). A more focused analysis of the distribution of variation between the different regions found similar levels (73–74 %) of within accession variation in the East and West group. The greatest within-population variation was found in the North group, at 76 %, and the least variation in the South (69 %). The regional Phi_PT_ values reflect the between-population variation and indicate that this is highest in among accessions in the Southern group.

**Table 2:** Summary of diversity described in European ecotypes by AMOVA

Source	df	SS	MS	Est. var.	%
Between regions	3	48,323	16,108	85	8
Between accessions	86	230,273	2678	247	24
Between individuals	626	446,237	713	713	68
Total	715	724,833		1045	100

Stat.	Value	*P*(rand ≥ data)
Phi_RT_	0.081	0.001
Phi_PR_	0.257	0.001
Phi_PT_	0.318	0.001

Region	No. of individuals	No. of accessions	Between accessions (%)	Within accessions (%)	Phi_PT_	*P* value
“East”	96	12	27	73	0.268	0.001
“North”	360	45	24	76	0.236	0.001
“South”	124	16	31	69	0.310	0.001
“West”	136	17	26	74	0.260	0.001

*Df* degrees of freedom; SS sum of squares, *MS* mean square, *Est var.* estimated variance. Phi_RT_ = AR/(WP + AP + AR) = AR/TOT among regions. ${\text{Phi}}_{\text{PT}} = \left( {{\text{AP}} + {\text{AR}}} \right)/\left( {{\text{WP}} + {\text{AP}} + {\text{AR}}} \right) = \left( {{\text{AP}} + {\text{AR}}} \right) / {\text{TOT}}$ (among individuals within accessions). ${\text{Phi}}_{\text{PR}} = {\text{Phi}}_{\text{RT}} + {\text{Phi}}_{\text{PR}} = {\text{AP/WP}} + {\text{AP}}$ (among accessions within region) where AR is between regions; AP is between accessions within region; WP is between individuals within accession

Individuals divided into four regions as described by PCA (Fig. 1 and defined in Supplementary Fig. 1). Regions described as “North”—upper quadrant of PCA plot including individuals from the UK, Northern Europe and Spanish outliers. “West”—bottom left group comprised of individuals from Spanish and Portuguese sample sites. “South”—bottom quadrant comprising Italian, Bulgarian and Turkish individuals. “East” group describes individuals on centre right of PCA plot, including Romania, Czech Republic, Bulgaria

### Population structure in European *Lolium perenne* ecotypes

To understand the broad genetic diversity and distribution across Europe, unbiased PCA was performed on the allele frequency for each of the 2185 SNPs within each of the 90 sample locations (accession) (Fig. 1a). PCA uses no prior information on the genotypes in construction of the plot, but despite this the observed distribution bears a striking resemblance to the geographic distribution of the original sampling sites. An East–West distribution was observed on PC1, in addition to a strong UK and Iberian divide on PC2. A strong correlation (*R*^2^) of 0.798 was found for PC1 to longitude (*P* < 0.001) and 0.712 for PC2 to latitude (*P* < 0.001) (Fig. 1b, c). Significant correlations were also observed between altitude and PC2 (−0.347, *P* < 0.001). Ecotypes from the UK were found to cluster in the upper left quadrant of the PCA plot, with particular similarity of accessions originating from England and Ireland. Accessions from Scottish islands and Wales were more divergent. A strong Iberian cluster was observed, with exception of one Portuguese accession (PT4; Ba13132) and the inclusion of an Italian accession (IT6; Ba13470). The centre of the PCA plot shows divergence of accessions along PC2 approximately split by the Alps Mountain range. Accessions originating from Eastern Europe are found on the right hand side of the plot, with particular extremity shown by those collected from Bulgaria.

**Fig. 2:**
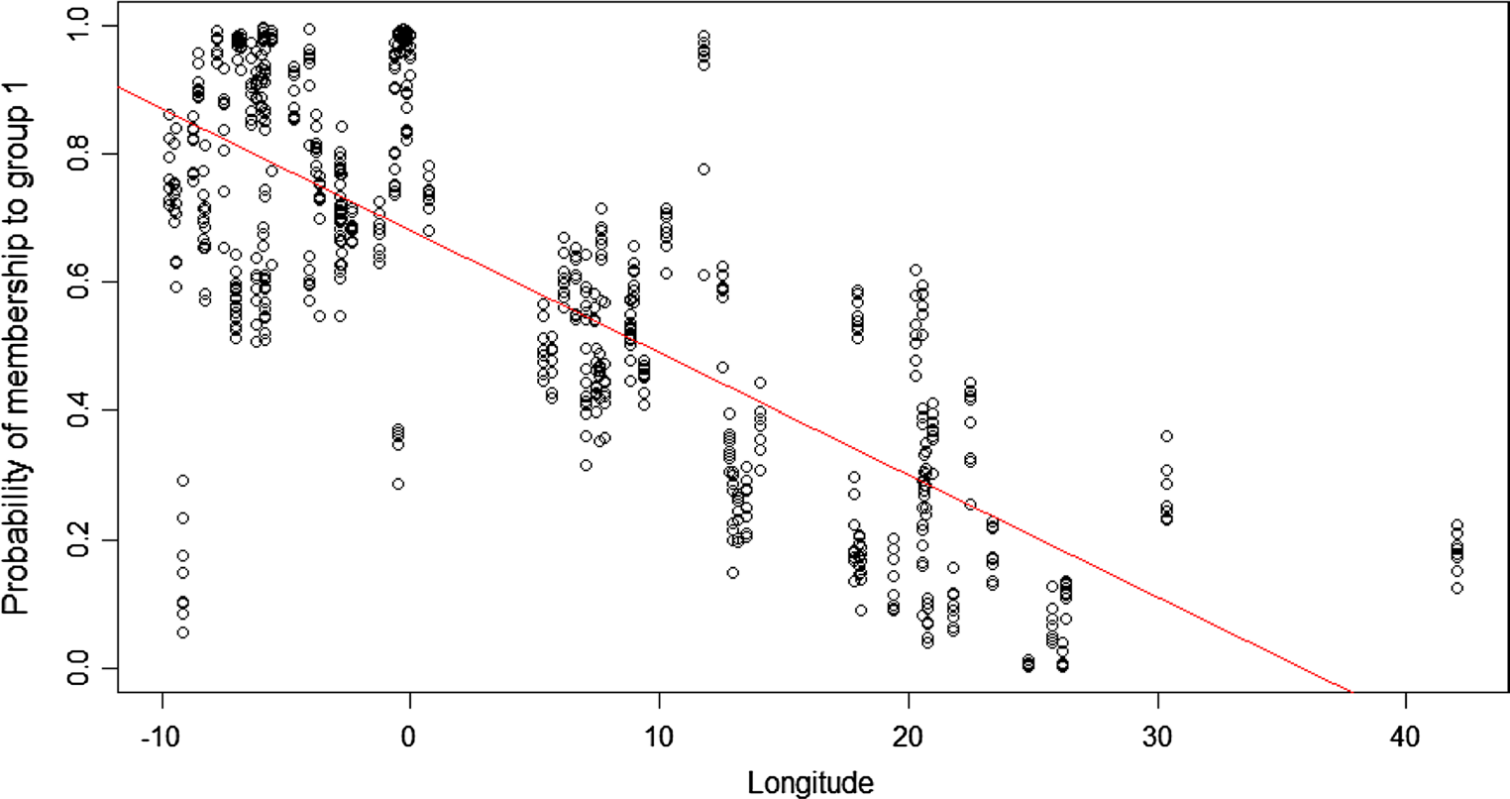
STRUCTURE analysis of ecotypes based on 2185 SNPs for *K* = 2, with probability of membership to group 1 shown against longitude of sample site. Optimal value of *K* determined using STRUCTURE Harvester. The consensus probability of group membership, as determined using CLUMPP, is plotted against the longitude of the sample site for each individual. Correlation of longitude to group 1 membership; *R* ^2^ = −0.7815

The population structure of the European ecotypes was also examined using STRUCTURE. The optimal number of subgroups (K) within this large collection of individuals was found using Structure Harvester to be two (Supplementary Fig. 2). These data are presented as a scatterplot of individual genotype probability of membership to group plotted against the longitude of the sample site (Fig. 2). In agreement with the PCA, there was significant strong correlation of probability of group membership to longitude (*R*^2^ = 0.782, *P* < 0.001). The notable outlier (small probability of group 1 membership and low longitude value) was PT4, which was expected given the clustering in the PCA plot (Fig. 1). A small secondary peak was also observed at 4 subgroups (Supplementary Fig. 2). Two of these groups had significant correlation to longitude (*R*^2^ = 0.766, *P* < 0.001) and latitude (*R*^2^ = 0.737, *P* < 0.001). The probability of an individual’s group membership was averaged for each region, as defined by the PCA plot (Supplementary Fig. 1). High probabilities were found for each group (group 1, average probability to South region of 0.57; group 2 to North region of 0.59; group 3 to East region of 0.72; group 4 to West region of 0.80) suggesting that the secondary peak at *K* = 4 was reflective of the PCA plot.

### Identification of primary genetic–geographic markers

To identify the markers contributing to the most prominent genetic structure and variation, the top 50 markers (as determined by their loading) for PC1 and PC2 were identified (Tables 3, 4). Markers within the same contig were commonly seen to have a similar rank within a principal component. This occurred for contigs 35543, 40624 and 7394 in the top 50 of PC1 and for 7 contigs in the top 50 markers contributing to PC2, indicative of closely linked markers behaving similarly as would be expected for robust array SNP probes. The BLASTx annotation for the contigs in which these markers were located was then assessed to determine if putative adaptive transcripts could be identified. Further showing that transcript/loading associations were robust, we observed that several contigs had the same annotation: for example, contigs 35,543 and 40,624 (Table 3) both returned a hit to formate-tetrahydrofolate ligase, and this was represented by 6 markers in the top 50 for PC1. A similar occurrence was seen for PC2 with an aarF domain containing protein kinase identified as a best hit from 3 markers contained in 2 contigs (7729 and 49,805).

**Table 3:** Top 50 markers contributing to the loading on PC1

Marker	PC1	BLAST
Contig33338_1322	0.049	Major facilitator superfamily domain-containing protein 5-like
Contig53271_67	0.046	NA
Contig49873_302	0.046	Histidine-containing phosphotransfer protein 2
Contig35543_1175	0.045	Formate-tetrahydrofolate ligase-like
Contig17494_1000	−0.044	Glucan endo-1_3-beta-glucosidase GV
Contig34149_1405	0.044	PP
Contig42055_156	−0.044	PP
Contig40624_321	0.043	Formate-tetrahydrofolate ligase-like
Contig40624_549	−0.043	Formate-tetrahydrofolate ligase-like
Contig7394_1265	−0.043	66 kDa stress protein-like
Contig7394_878	−0.042	66 kDa stress protein-like
Contig35543_365	0.042	Formate-tetrahydrofolate ligase-like
Contig32759_449	−0.040	Protein RER1B-like
Contig50617_428	−0.040	Reticulon-like protein B8-like
Contig8691_750	−0.040	PP
Contig9865_491	0.040	PP
Contig52098_89	−0.040	Farnesylated protein 2 [*Hordeum vulgare* subsp. *vulgare*]
Contig43816_303	−0.040	Hypothetical protein SORBIDRAFT
Contig40661_72	−0.040	GSK-like kinase [*Triticum aestivum*]
Contig36221_1388	0.040	PP
Contig10390_1015	−0.039	PP
Contig37988_1071	−0.039	PP
Contig6836_1026	−0.039	Malonyl-CoA-acyl carrier protein transacylase_mitochondrial-like
Contig7723_139	−0.039	Methionine aminopeptidase 1A-like
Contig43968_560	−0.039	cycloartenol synthase
Contig31420_213	−0.039	Importin subunit alpha-1a-like
Contig16521_537	−0.038	Laccase LAC5-4 [*Lolium perenne*]
Contig40828_1699	−0.038	NPH1-2
Contig6666_679	0.038	PP
Contig45469_388	0.038	PP
Contig32047_1499	0.038	PP
Contig10442_410	−0.038	PP
Contig6744_927	−0.037	Adenylyl cyclase-associated protein-like
Contig50264_1001	−0.037	PP
Contig32202_682	−0.037	PP
Contig15977_1173	−0.036	Chromosome-associated kinesin KIF4-like
Contig6744_417	−0.036	Adenylyl cyclase-associated protein-like
Contig35543_281	−0.036	Formate-tetrahydrofolate ligase-like
Contig35543_641	−0.036	Formate-tetrahydrofolate ligase-like
Contig36358_326	−0.036	PP
Contig7673_909	−0.036	PP
Contig7054_239	0.036	PP
Contig49944_954	−0.036	Gamma-tocopherol methyl transferase [*Triticum aestivum*]
Contig41068_335	0.035	PP
Contig20240_138	0.035	PP
Contig31682_1562	−0.035	BEL1-like homeodomain protein 6-like
Contig51006_260	−0.035	Ribosome biogenesis protein NSA2 homologue
Contig50500_372	−0.035	PP
Contig51969_109	−0.035	PP
Contig51913_371	0.035	PP

*NA* not available (no BLAST result), *PP* predicted protein

**Table 4:** Top 50 markers contributing to the loading on PC2

Marker	PC2	BLAST
Contig31170_2438	−0.057	PP
Contig17179_1421	−0.057	Cysteine proteinase 1-like
Contig31170_1515	−0.056	PP
Contig40677_129	−0.052	Delta(24)-sterol reductase-like
Contig31170_798	0.052	PP
Contig49805_880	−0.051	Uncharacterized aarF domain-containing protein kinase
Contig40677_1150	−0.050	Delta(24)-sterol reductase-like
Contig6946_62	−0.049	PP
Contig52497_155	−0.049	NA
Contig7729_241	−0.047	Uncharacterized aarF domain-containing protein kinase
Contig6714_427	0.046	PP
Contig9365_585	−0.045	PP
Contig41380_775	−0.045	Putative cytochrome P450 [*Lolium rigidum*]
Contig40621_1539	−0.043	PP
Contig7958_544	0.043	Isoflavone reductase
Contig16471_106	0.043	PP
Contig49805_127	−0.041	Uncharacterized aarF domain-containing protein kinase
Contig6632_2269	0.041	PP
Contig31122_190	0.040	PP
Contig6914_70	0.040	PP
Contig31167_1317	−0.040	Myosin-J heavy chain-like
Contig32310_158	−0.040	Pyruvate dehydrogenase E1 component subunit alpha-like
Contig35649_435	0.039	Trehalose-6-phosphate synthase
Contig44219_328	0.039	PP
Contig50225_614	0.038	Zinc finger CCCH domain-containing protein 49-like
Contig35863_78	−0.037	Acyl-coenzyme A oxidase 3_peroxisomal-like
Contig6855_1933	−0.037	PP
Contig8527_579	0.037	PP
Contig31167_864	−0.037	Myosin-J heavy chain-like
Contig37468_683	0.037	CBL-interacting protein kinase 2-like
Contig52209_230	0.037	Putative cytochrome P450 [*Lolium rigidum*]
Contig31242_1073	−0.037	Hydroquinone glucosyltransferase_putative_expressed
Contig49802_385	−0.036	Transcription factor Pur-alpha 1-like
Contig9232_399	0.036	PP
Contig52297_289	−0.036	PP
Contig40957_743	−0.036	Annexin D5-like
Contig40660_160	−0.036	Cell division cycle protein 48_putative_expressed
Contig18648_66	−0.036	NA
Contig50193_695	−0.036	PP
Contig42143_365	0.036	NA
Contig53284_164	0.036	PP
Contig41676_341	−0.035	PP
Contig6632_1814	0.035	PP
Contig50143_1456	−0.035	Delta-1-pyrroline-5-carboxylate synthase-like
Contig35084_2381	−0.035	Villin-2-like isoform 1
Contig7244_936	−0.035	PP
Contig13040_111	−0.035	PP
Contig7101_762	−0.035	Hypothetical protein SORBIDRAFT_10g007850
Contig7394_1265	0.035	66 kDa stress protein-like
Contig7394_878	0.035	66 kDa stress protein-like

*NA* not available (no BLAST result), *PP* predicted protein

## Discussion

### Creation of an SNP resource for *Lolium perenne*

Based on NGS transcriptome sequencing, we have created a publically available resource of 2185 high-quality genetic markers which can be used for rapid genotyping of *L. perenne.* This significantly increases the number of SNPs assayed on a single array from the previously published 768-plex Illumina GoldenGate array (Studer et al. 2012), which are complementary with our marker set (a v2 assay is being developed with many of these SNPs included). The assay described in this paper provides a new resource to elucidate the selective forces operating on the genomes of naturally occurring perennial ryegrass. A better understanding of these evolutionary forces will have implications for the development of new resilient grassland systems in the context of climate smart agriculture. A publically available SNP genotyping resource will also enable a population-based approach to conservation genetics and higher resolution study of the population structure of *L. perenne*. Conversion of NGS transcriptome sequence variants into validated SNP probes was ~64 % successful, which appears to be consistent with similar assays based on NGS data (van Bers et al. 2012; Verde et al. 2012). Given the de novo nature of this transcriptome assembly, the heightened stringency measures taken in selecting putative SNPs was indeed necessary and, if repeated, could now take into account the existence of recent, more in-depth NGS assemblies such as the annotated transcriptome of Ruttink et al. (2013) or the draft *L. perenne* genome currently in progress. Regardless, the assay represents a significant increase in SNP resources for *Lolium* and highlights the value of developing fixed platforms which can be used to assay the same markers across a broad range of material.

### Population structure of *Lolium perenne* across Europe

This study reveals the genetic structure of European *L. perenne* populations and demonstrates strong correlations between genotypes and geographic origin despite no prior knowledge. Previous studies on *L. perenne* have reported a population structure (Skot et al. 2005; Yu et al. 2011; Bolaric et al. 2005a, b; McGrath et al. 2007; Balfourier et al. 1998, 2000). Balfourier et al. (1998, 2000) reported an association of geographic origin to genetic diversity, initially via 120 populations but only across 12 loci marker set and then from 28 populations using cpDNA identifying 15 haplotypes. Similar results were reported by McGrath et al. (2007). However, the link to geography has not been as clearly defined as in this study. Our results provide a greater resolution as a consequence of a larger marker set and sample size. Similar genetic–geographic correlations have been seen previously across Europe in >3000 human genotypes with a high density (500 k) SNP array (Novembre et al. 2008). Substructuring of *L. perenne* populations due to geography may be indicative of either adaptation to different ecological habitats, or due to changes in allele frequency resulting from population subdivision (i.e. isolations by distance and/or from glacial refugia): potentially a mixture of both. Divisions of the *L. perenne* population across both latitudinal and longitudinal gradients have been proposed previously in limited sample population sizes and with a reduced marker set using isozyme analysis (Balfourier et al. 1998) and chloroplast DNA haplotyping (Balfourier et al. 2000). *L. perenne* has been suggested to have arisen in the Middle East and subsequently migrated to Europe, with the Alps acting as a barrier to gene flow between North and South Europe (Balfourier et al. 1998, 2000). However, this scenario would be expected to result in a diversity gradient from West to East due to the sequential sampling of allele frequencies from the wave of advance and result in lower diversity in the Western regions. This study, however, found comparable diversity between accessions in East and West regions (Table 2), which does not support this theory.

The alternative scenario is one of the repeated population expansion and contraction due to periodic glacial cover, in which *L. perenne* populations were forced back to Western, Eastern and Central refugia along the Mediterranean prior to subsequent re-expansion to Northern latitudes. Populations in each refugium diverge during the glacial maxima and then interact with divergent allele frequencies mixing in areas of expansion overlap, resulting in clines that run approximately East to West. Our study supports this scenario due to comparable diversity in East and West regions, and greater diversity in the Central/Southern region and lowest between accession diversity in the North as indicated by Phi_(PT)_ (Table 2).

Evidence has also been previously provided to support the migration from South to North Europe via comparisons of geographic groups using AMOVA, whereby no variation was found between Near Eastern and Southern European ecotypes, nor Western and Southern European ecotypes (McGrath et al. 2007). AMOVA on these data between neighbouring regions identified variation between all neighbouring populations (Supplementary Table 1) unlike the previous study. McGrath et al. (2007) also found no variation when comparing populations north and south of the Alps. In this study, despite the close geographic proximity of some accessions in northern Italy and Switzerland, the genetic divide is disproportionately large, as observed from PCA, supporting the theory of a physical population barrier dictated by the altitude of the Alps. The differing results are probably a reflection of number of markers used and number of sample populations used, together these have given a greater resolution of genetic diversity and association with geography.

Two ecotypes, PT4 and IT6, were found to be outliers based on their genotypes, compared to their geographic origin (Fig. 1). Their actual geographic sample site was found to be of low altitude and coastal. Therefore, it is proposed that these ecotypes may have been transported via (sea) trade routes from their “genetic” origin to their current geographic location. PT4 (Ba13132) has previously found to be genetically outlying from other *L. perenne* Portuguese accessions based on AFLP analysis (Cresswell et al. 2001), supporting the results in this study. This suggests that the resolution of the SNP resource offers the potential to distinguish recent migrations due to human activity from those undergone as the species spread from refugia.

This study, as one of creation, validation and investigation of *L. perenne* ecotype diversity, has been able to unexpectedly provide a greater resolution of the European colonisation of perennial ryegrass, which deserves further and more detailed analysis. This, coupled to chloroplast data, may answer some of the questions regarding the migration history of *L. perenne* raised by Balfourier et al. (1998, 2000).

### Diversity of ecotypes

The greatest proportion of the variation identified in this large ecotype collection was found between individual plants (Table 2). This is not unexpected due to the outbreeding nature and the self-incompatibility complex in *L. perenne* (Thorogood et al. 1993). It is also comparable to between individual variation of 61 and 82 % previously found in European and Irish *L. perenne* ecotypes, respectively, based on cytoplasmic markers across 78 accessions (McGrath et al. 2007). Bolaric et al. (2005b) also reported 68 % within European cultivars and 74 % within Polish ecotypes.

### Identification of markers contributing to geographic division

A number of markers within the same contigs were identified as having a similar loading to a principal component, as would be expected in the case of genuine associations of genotype with geographic location. This was highlighted by examples in the top 50 markers for PC1 and PC2 in Tables 3 and 4, but was common through the rankings. Markers associated with the East–West divide (PC1) are listed in Table 3. To identify transcripts putatively associated with the geographic split and thus possibly adaptive variation, the BLASTx annotations of the RNAseq contigs from which these markers were derived were investigated further. Whilst a majority of the sequences returned no hit or hits to predicted proteins only, several contigs were identified which may be indicative of adaptation to environment. These included six SNPs across two contigs having a greatest similarity to formate tetrahydrofolate ligase, which has been associated with CO_2_ metabolism (Dupont 2008) and to photorespirational response to stress (Cai et al. 2011). Subsequent analysis has demonstrated that these two contigs are actually the same transcript, overlapping by 20 bases (which would have been insufficient for contig merging in the assembly parameters used here). Reassembly of contigs to a draft *L. perenne* genome sequence is ongoing and annotations will be updated accordingly. Several other transcripts showed multiple markers associated with the PC1 divide: adenylyl cyclase-associated protein and a 66 kDa stress-related protein both had two SNPs within the same contig present in the top 50 markers. The former of these has been associated with auxin-regulated cell proliferation (Ichikawa et al. 1997) and also in blue light signalling (Iseki et al. 2002)—another marker in a transcript encoding NPH1-2 is associated with blue light response (Sakai et al. 2011). The 66 kDa stress-related protein has a WD40 functional domain, which has been linked to developmental signalling pathways in plants (van Nocker and Ludwig 2003). Interestingly, the same markers within this latter transcript also appear in the top 50 contributing to PC2, suggesting a strong association of the transcript with geographic diversity. Other transcripts associated with developmental pathways and/or stress responses are also observed to contribute to PC1: histidine-containing phosphotransfer protein 2 has been demonstrated to play a role in cytokinin signalling in *Arabidopsis* (Hutchison et al. 2006), whilst GSK-like kinases are known to be involved in multiple developmental and stress signalling pathways in plants (Choe et al. 2002). A transcript encoding gamma-tocopherol methyl transferase was also identified: tocopherols are essential micronutrients in plants and act to protect against oxidative stress (Koch et al. 2003). Several transcripts involved in import/export are also identified (major facilitator superfamily protein, importin subunit), along with transcripts involved in cell wall lignification (laccase).

Markers contributing to PC2, or the North–South axis, tell a similar story. Markers were identified in transcripts linked to plant growth/development: cysteine proteinase (Grudkowska and Zagdanska 2004), Pur-alpha transcription factor (a general regulator of cell cycle gene expression; Trémousaygue et al. 2003) and a cell division cycle protein. Stress-related transcripts are also identified, including the same 66 kDa protein as for PC1. Two markers are identified in a contig encoding an aarF/ABC1 domain protein: interestingly, this family of proteins has been implicated in tocopherol biosynthesis, a process also putatively affected in PC1 and a possible response to oxidative stress (Martinis et al. 2013). A transcript encoding delta(24)-sterol reductase was also identified: again, sterols play a role in antioxidant activity in plants (Wang et al. 2012) and cycloartenol synthase (involved in the production of sterol intermediates) was also identified on PC1. Hydroquinone glucosyltransferase was also identified on PC2 and phenolic hydroquinones also have antioxidant properties, suggesting a putative general role for these compounds in plant adaptation. It should be noted that sterol levels also play a role in cold tolerance in plants (Palta et al. 1993), and we also observe a marker in a transcript encoding a trehalose-6-phosphate synthase, which is also implicated in cold tolerance (Li et al. 2011). Finally, and distinct to the observations for PC1, several transcripts were observed to be involved in cytoskeletal development (myosin, villin) and Ca^2+^-mediated signalling (CBL-interacting protein, annexin).

The pathways identified on both PC1 and PC2 are all strong candidates for adaptational responses to environment. However, further research will be needed to see if specific haplotypes are indeed associated with phenotypic changes that would suggest adaptation. It is also possible that these markers represent founder effects as the *L. perenne* subpopulations spread from refugia.

### Potential applications of the iSelect assay

This analysis, based on genome-wide nuclear marker technology, improves the resolution with which the population substructure can be assessed, offering a clearer understanding of how the migration of *L. perenne* across Europe may have occurred. In addition, there is potential to identify the genomic regions strongly differentiating the different subpopulations and thus untangling the effects of migration and adaptation. This latter point is of particular importance in the face of global issues of climate change and food security—if the geographic correlations observed are tied to ecological habitat, then genomic regions can potentially be identified that are involved in local adaptation which can be mined for useful traits needed in *L. perenne* breeding programmes (and possibly used for gene discovery in other grasses). The next steps will be to identify the extent of linkage disequilibrium within *L. perenne* to determine the power of this marker set to perform genome-wide association studies (GWAS) of adaptive traits, as well as to determine the amount of ecological diversity which has been captured within existing breeding programmes. Large-scale genotyping has the potential to significantly improve the rationale of conservation, characterisation and utilisation of crop genetic resources (McCouch et al. 2012). In the case of perennial ryegrass, the iSelect array developed in this study can potentially be used to explore existing variation in ryegrass collections, manage seed multiplication and enhance quality control procedures. This assay also provides a means to identify core collections for ryegrass ecotypes for multi-environment field testing to identify candidate genes underlying quantitative traits responsible for adaptation to changing climatic conditions.

## Conclusion

This publically available resource significantly expands on the marker density previously available for genotyping the agriculturally important forage crop species, *L. perenne.* The validated markers have allowed a greater resolution of the genetic–geographic population structure and diversity available in the ecotypic population in Europe. These populations, along with the array, will provide a mechanism to identify the markers, genes and traits to respond to the demands of a rapidly changing climate.

### Author contribution statement

WP & MH designed the research. MH conducted the NGS analysis, SNP discovery and assay design. TB conducted genotyping and genetic diversity analyses. RM advised on population genetics analysis. TB, MH & WP wrote the paper. RM provided critical review of the paper. IT collected, stored and catalogued germplasm for the ecotype collection.

## Electronic supplementary material

Supplementary material 1 (EPS 8409 kb)

Supplementary material 2 (EPS 1829 kb)

Supplementary material 3 (XLSX 229 kb)
